# Getting the Perpetrator Incorporated and Prioritized in Homicide Investigations: The Development and Evaluation of a Case-Specific Element Library (C-SEL)

**DOI:** 10.3390/ijerph17176430

**Published:** 2020-09-03

**Authors:** August Daniel Sutmuller, Marielle den Hengst, Ana Isabel Barros, Pieter van Gelder

**Affiliations:** 1 Values, Technology and Innovation, Safety and Security Science, Faculty of Technology, Policy and Management, Delft University of Technology, 2628 BX Delft, The Netherlands; p.h.a.j.m.vangelder@tudelft.nl; 2 RTI Laboratories, Dutch Police, 3709 JA Zeist, The Netherlands; marielle.den.hengst@politie.nl; 3 TNO Defense, Safety and Security, 2597 AK The Hague, The Netherlands; ana.barros@tno.nl

**Keywords:** person of interest, homicide, criminal investigation, prioritization, incorporation, perpetrator, C-SEL

## Abstract

Homicide investigators in the digital era have access to an increasing amount of data and the processing of all persons of interest and pieces of evidence has become nearly impossible. This paper describes the development and evaluation of a case-specific element library (C-SEL) that can be used to incorporate and prioritize persons of interest in homicide investigations. In a survey, 107 experts in the field of criminal investigation assigned an initial score to the elements. Each element was extended with underlying factors that can be used to adjust the initial score based on the relevance and credibility of the source. A case study was conducted using three Dutch real-world cases to evaluate the methodology. The results look promising and are better than four methodologies currently used in practice.

## 1. Introduction

The United Nations Office on Drugs and Crime [1] estimated, in their latest global study of homicide, that in 2017, approximately 464,000 deaths were due to intentional homicide. Homicides have a large impact not only at a personal and family level, but also yield a financial burden to society. DeLisi et al. showed [2] that homicides yielded higher costs than other crimes such as armed robbery. Globally, the homicide rate has dropped over the last three decades, but at the same time, the percentage of unsolved homicide cases has increased in many countries [3,4,5,6,7]. Over these decades, our environment has changed from analogue to digital. Homicide investigators now have access to an increasing amount of data, making it possible to incorporate and collect an increasing number of persons of interest and pieces of evidence. This could contribute to the incorporation of the perpetrator into the investigation and the clearance of the case, but the processing of all persons of interest and pieces of evidence becomes an ever-more daunting task. The increasing availability of data is one of the challenges the police are now facing in community policing, emergency response, and also in homicide investigations. Information overload is accompanied by excessive time demands on detectives to make sense of information, which leads to systematic delays in processing intelligence or evidence [8]. The volume of information that is generated in investigations can be seen as one of the reasons that cases in the future will become cold cases [9]. There is currently a lack of methodologies and tools to support homicide investigations that can cope with the increasing volume of information available.

Therefore, the aim of this paper was to introduce a novel methodology that assists homicide investigators with the incorporation and prioritization of persons of interest in today’s world. To deal with the incorporation and prioritization of persons of interest in this digital era, we adopted the idea of using a library of elements to design case-specific prioritization tools [10]. In his book, Wilson [10] provides guidelines on how an investigative team can select and value elements in their case. The subjective nature of the valuation of elements is seen as a weakness of this methodology [11]. To overcome this weakness, a fixed number of elements is selected, for which an initial score is established and underlying factors are determined. These factors allow elements to be adjustable, making the methodology case-specific. In general, some elements or pieces of evidence will have more value compared to others in identifying the perpetrator (initial score). However, some elements could be important in one case and useless in another (underlying factors). Broeders [12] gives an apt description of this topic: “apart from determining the source of the trace, establishing an association between the trace and the incident under investigation is of vital importance for the relevance of the evidence to the central investigative question”. In order to evaluate the effectiveness of the introduced methodology and the proposed case-specific element library (C-SEL), the new methodology was evaluated on two performance measures and compared with four currently used methodologies in a case study. Finally, conclusions and directions for further research are drawn.

## 2. Background

### 2.1. Management of Homicide Investigations

The management of homicide investigations shows subtle differences between countries, but manuals regularly show a three-phased process [13,14]. The three phases can be separated by time and purpose. The collection of evidence, the identification of the victim, and the incorporation of possible suspects are central to the first phase. In the second phase, decisions are made about what lines of enquiry or persons of interest should be prioritized based on analytical products. In the last phase, a case file has to be produced, proving the guilt of the suspect, and eliminating all alternatives. The digital era, with access to unlimited amounts of data and the development of diverse new pieces of evidence, mainly influences the first two phases of homicide investigations. Therefore, this research focused on the collection and prioritization phases of homicide investigations.

### 2.2. Currently Used Methodologies

In the literature, four analytical products or methodologies were found that assist criminal investigators with the collection and prioritization of (large numbers of) persons of interest by the weighting of case-specific elements or pieces of evidence. A brief outline is given on how these four methodologies function.

Thousands of persons of interest and a lack of capacity in a Canadian high-profile case were the start of a prioritizing tool named the Person of Interest Priority Assessment Tool (POIPAT) [10]. The methodology uses elements (like access to the crime scene or physical characteristics) to construct a case-specific tool. Weight is assigned to the elements using a benchmark element. The weighted elements are then used to prioritize persons of interest. In each case, a tool is constructed and weighted by the investigative team.

Trace Investigate Evaluate (TIE) was developed after the evaluation of the Yorkshire ripper case, where the lack of central administration prevented the perpetrator from being detected. TIE is used in investigations where the perpetrator is not caught committing the crime. The methodology uses categories to collect groups of people (like people having access to the crime scene or matching physical characteristics). After the persons of interest are incorporated, behavioral investigative analysts assign weights to elements in order to prioritize between the persons of interest [13].

Rasterfahndung was developed in order to identify groups of potential terrorists in the 1970s [15], but in the last decade, this form of reactive dragnet has been used in homicide investigations by the police of Nordrhein-Westfalen. People from a geographical area are incorporated into the investigation, based on case-specific leads (like telephone sites or witness statements about transportation). Based on other leads (like physical characteristics, age, criminal history, and shoe size) the persons of interest are prioritized. Multiple pairwise comparisons by team members are used to assign weights to the leads [16].

The Analysis of Competing Hypotheses (ACH) was designed to analyze difficult problems and to overcome analytical and cognitive pitfalls [17]. The methodology has been adopted by Dutch crime analysts to prioritize between persons of interest. A matrix of hypotheses (the persons of interest) by arguments (pieces of evidence) is constructed and the inconsistency between the hypotheses and pieces of evidence is scored. A ranking is made between persons of interest based on pieces of evidence that are inconsistent with the person of interest. 

Sutmuller, den Hengst, Barros, and van Gelder [18] provided a comparison between these four methodologies that are currently being used in homicide investigations. The four methodologies were evaluated across three recently solved Dutch homicide cases using the following questions: can the methodology incorporate the name of the perpetrator into the investigation within the first two weeks (collection) and can the methodology prioritize the perpetrator within the top ten percent of persons of interest (prioritize)? The methodologies showed comparable and modest results. All methodologies failed to meet the performance criterion for collection and prioritization in one of the three cases [18]. In addition, Sutmuller et al. [18] showed that a large number of persons of interest, tens of thousands, would be incorporated using the methodologies and stated that it is important to take into account the reliability and credibility of the source of evidence [18].

## 3. Materials and Methods: The Development of C-SEL

In this section, we developed a methodology (C-SEL) that assists homicide investigators with the incorporation and prioritization of persons of interest. First, elements for the library were selected based on literature and personal experience. Second, the selected elements were valuated through a survey conducted with three groups of experts working in homicide investigation. Third, underlying factors were determined to enable the elements to become adjustable. The section ends with a walk-through of C-SEL.

### 3.1. Selection of Elements

In the literature, two extensive reviews were found on factors influencing the clearance of homicide investigations [19,20]. Although many factors are discussed, most of them are beyond the control of homicide investigations (like the gender or race of the victim). One class of factors, case dynamics, is under the control of homicide investigators. Case dynamics are factors that affect the development of the subsequent investigation once the police become aware of a homicide [20]. Examples of case dynamics are the number of witnesses or the types of evidence found at the crime scene. Understanding the correlation between certain case dynamics and case clearance will be the starting point for selecting elements for the library.

#### 3.1.1. Case Dynamics

In the literature on case dynamic factors, there is only one factor that, over many studies, shows a positive relation between its presence and case clearance. This factor is the presence of eyewitnesses [21,22,23,24,25].

In more recent years, research has focused on the contribution of forensic evidence to case clearance. The role of physical evidence, collected at the initial crime scene, in moving investigations toward the identification of homicide suspects is complex and poorly understood [26]. For example, the availability of DNA showed mixed results over studies. McEwen and Regoeczi found that DNA evidence showed a negative effect on case closure [23]. Schroeder and White found that cases in which DNA was available, before the arrest of a suspect, were associated with a longer time to clearance [24]. It should be noted that the longer clearance time can be due to an unknown offender (whose DNA registration is not yet available in a system like the Combined DNA Index System (CODIS)). Recently, Wells et al. [27] analyzed the impact of DNA evidence in the judicial process for sexual crimes (specifically, the use of older sexual assault kits that had not been tested yet). They argued that the growth of the CODIS database will increase the effectiveness of the judicial process for sexual crimes. For property crimes, when DNA evidence was included in investigations, twice as many suspects were identified [28]. The research in this area is far from complete. The limited access for researchers to police data has resulted in few studies that included forensic evidence and its possible contribution to homicide investigations [23].

Studies that investigated the contribution of digital evidence, an important topic considering the rapidly increasing amount of digital data available, are even more scarce. Weidman states that video surveillance had a decidedly larger impact than DNA in solving homicides [29]. Roycroft found that phone analysis contributed by 25% and closed-circuit television (CCTV) by 21.7% to the solution of cases [30]. These are indications that digital evidence contributes to case clearance, but a comprehensive overview of the types of digital evidence and their contributions has yet to be conducted.

In the literature, no comprehensive overview of case dynamic factors was found that could be used to select elements for our library. However, in recent qualitative research, detectives described cases as “difficult to solve” when lacking physical or trace evidence (such as DNA, CCTV, fingerprint, ballistic, or other trace evidence) and/or witness evidence [8]. Elements dealing with witness statements, physical evidence, and digital evidence will be included in the library.

#### 3.1.2. Structure of the Library

To provide structure to the library, the opportunity–motive–means (OMM) topology was used. In criminal law, the opportunity, motive, and means of a suspect are evaluated to reach a verdict about guilt. In handbooks on criminal investigations, this topology is used to explain how evidence can be structured [31,32]. This topology ensures a balance between the different perspectives and as such, opportunity is as relevant as motive or means. In addition, a distinction is made between group level and individual level. The group level elements are used to incorporate groups of persons of interest and to provide a first ranking. The individual level elements are used to further prioritize the persons of interest relative to each other. The OMM topology including the distinction between group and individual level elements, combined with both the literature and more than a decade of personal experience in criminal investigation resulted in a proposed library. The proposed library including the description of the selected elements is displayed in Table 1.

### 3.2. Valuation of the Elements

The selection of the elements was based on the literature and personal experience. Some elements will have more value in identifying the perpetrator compared to others; therefore, a survey was conducted with three groups of experts working in homicide investigations. After the elements were valued, possible double count of and dependency between elements in the library were addressed.

#### 3.2.1. Participants

Three groups of experts, all working for the Dutch Police, were invited to participate. The first group consisted of Crime Analysts (N = 37; Mean age = 44.58; Mean years of experience = 18.81)—crime analysts of the Dutch Police are trained to steer criminal investigations based on information. The second group consisted of police officers either pursuing or possessing a Masters of Criminal Investigation (N = 42; Mean age = 41.07; Mean years of experience = 16.31)—a group of highly-educated police officers that are recruited to improve the quality of criminal investigation. The last group consisted of Investigative Psychologists (N = 28; Mean age = 42.37; Mean years of experience = 13.68)—this group is responsible for a range of products such as behavioral case analyses. A total of 231 experts were invited by email to complete the survey; in total, 115 experts started the survey and 107 experts completed the survey. The response rate for this survey was 46.32%. Of the 107 participants, three participants chose to leave no or partial demographic information about themselves.

#### 3.2.2. Survey

The participants were instructed to sort the elements from most important to least important. The first item was about the main categories and stated: “A murderer must have the opportunity, motive and the means to commit the crime. Sort the categories from most to least important”. When the first question was sorted, two questions per main category were presented, the first on group level (Which group of people would you investigate first?) and the second on individual level (Which person is the most interesting?). The categories of the first question and the elements of the six subsequent questions were randomly displayed among participants. In the eighth question, some demographic information about the participants was asked to gain more insight into the backgrounds of the participants. This study was approved by the Human Research Ethics Committee of the TU Delft on 1 September 2020 (application number: 1268).

#### 3.2.3. Survey Results

There was an agreement between the three groups of experts (H(2) = 0.006, *p* = 0.997). No significant difference in the survey results was found between Crime Analysts (Mdn = 3.01) and Masters of Criminal Investigation (Mdn = 3.01), (U = 575, z = −0.037, *p* = 0.971), between Crime Analysts (Mdn = 3.01) and Investigative Psychologists (Mdn = 3.56), (U = 571, z = −0.092, *p* = 0.927), or between Investigative Psychologists (Mdn = 3.56) and Masters of Criminal Investigation (Mdn = 3.01), (U = 578, z = −0.006, *p* = 0.995). The similarity of results between experts supported us in determining initial scores for the elements. Due to the design, the most preferred element had the rank closest to one. As the most preferred element should hold the highest initial score, the rank was inverted through the calculation of an average rank score. After the average rank score of each question was determined, the scores were normalized by dividing them by the number of elements for that question. To calculate the initial score, the normalized rank scores were multiplied by the calculated average rank score of the main categories (Motive, 2.49; Opportunity, 2.08; Means, 1.44). In the last step, to convert to integers, the scores were multiplied by 100. The mean score and standard deviation of the survey results for the three expert groups as well as the calculated initial scores are displayed in Table 2.

#### 3.2.4. Double Counting and Dependency

When a methodology uses multiple criteria or elements to guide decision making (in this case the prioritization of persons of interest), it is important to address possible double counting of and dependencies between elements.

The problem of double-counting is caused by counting the same item twice. Based on a tendency of double counting, three revisions were made by the researchers. The first revision was the elimination of the elements of motive from the individual level. When a person of interest is incorporated in the investigation because he or she belongs to a group that has a motive, he or she will receive an initial score of 170 (GMo1), and he or she will automatically receive the initial score of 182 (IMo2) on the individual level. The same is true for GMo2 and Imo1: when a person of interest scores on GMo2, he or she will automatically score on Imo1. The group level elements remained included, because the element of motive can be used to select groups of persons of interest from police information or witness statements. The element also provides the opportunity to include persons of interest in a case with different motives or from different scenarios.

The second revision was the elimination of two means elements on the group level (GMe4 and GMe5). The elements were eliminated because there is a tendency to double count with two elements of means on the individual level (IMe8 and IMe7). The elements on the individual level were kept to enable comparative analysis of weapons, fibers, and tools for each person of interest; the group-level elements were eliminated, because it is difficult to incorporate groups of persons of interest based on having the same weapons, fibers, or tools as the perpetrator.

The last revision was to merge the elements dealing with the description of the perpetrator. The researchers state that merging the elements leads to a more elegant and efficient library. The three elements at the group level related to the source of the description (GMe1, GMe2, and GMe3) were merged with the six elements at the individual level related to description features of the perpetrator (IMe1, IMe2, IMe3, IMe4, IMe5, and IMe6). This leads to different initial scores for description features based on camera footage, witness statements, or offender profiles. A description based on camera footage (GMe2) received the highest initial score and we chose to set this as the benchmark (100%). The initial score of a description based on a victim statement (GMe1) received 60% of this benchmark and a description based on an offender profile (GMe3) received 57% of this benchmark. These percentages were used to recalculate the initial score of the features of the description. The elements that were included in the final version of the library are shown in Table 2.

A closely related aspect that had to be addressed was the dependency between elements (e.g., when someone’s telephone is at a certain location, the probability that the same person is seen by a witness at that location increases). In order to prevent this scoring bias, rules were developed to handle scoring on elements that showed some sort of dependency. When a person of interest scores on a combination of GO1, GO2, GO3, and GO4, the element with the highest score was used. The same logic was used for GMo1 and GMo2, and for IO1, IO2, IO3, IO4, and IO5. For the characteristics of a description (IMe1, IMe2, IMe3, IMe4, IMe5, and IMe6), the highest scoring source (GMe2, GMe1, and GMe3) was used.

Finally, because evidence of absence is not the same as the absence of evidence, 20% of the initial score was allocated to all elements of which the score had not (yet) been established.

### 3.3. Determination of Underlying Factors

A library was developed containing 24 elements. These 24 elements were provided with an initial score, based on the expert judgment of 107 experts working in the field of homicide investigation. In order to take the reliability and credibility of the sources of the elements into account, and to make the library case-specific, underlying factors were determined for each element in the library. A model of Anderson, Schum, and Twining [33] was adopted to adjust the initial score of the elements with underlying factors that were based on the relevance and credibility of the source. The model of Anderson et al. [33] was selected over more general approaches like the 4 × 4 grading system [34] because it was developed specifically to reason about evidence in criminal investigations.

#### 3.3.1. Relevance

The United States Rules of Evidence state that evidence is relevant if it has any tendency to make a fact more or less probable than it would be without the evidence. According to Anderson et al. [33], the relevance of evidence depends on whether the evidence is direct or indirect. Direct evidence holds only one reasoning step between the evidence and the matters revealed in the evidence. Indirect (or ancillary) evidence bears upon the strength or weakness in a chain of reasoning for evidence being argued as being directly relevant [33]. So, the first underlying factor of relevance is directness. One has to consider whether the evidence directly identifies the perpetrator. In order to take the situational aspect of a case into account, the uniqueness of the element within the case-specific population [10] was also included as an underlying factor. An element can be very unique or not unique at all (e.g., a man wearing pink jeans or a registered phone in the middle of the capital city). For elements of opportunity, it could be important to add the underlying factors of location and time. Weyermann and Ribaux [35] describe this as the unity of time, place, and action. The aim of an investigation is to place a suspect at the crime scene (location) at the time of the crime [35].

#### 3.3.2. Credibility

To determine the credibility of evidence, it is important to distinguish between tangible and testimonial evidence. This distinction is important, because the attributes of credibility for both sources are different [33]. The source level of the elements can be deduced from the description of the elements, six distinct sources were found: witnesses, police information, offender profiles, sensors, traces, and goods. Witnesses, police information, and offender profiles can be seen as testimonial (e.g., persons of whom a witness states that they were near the crime scene at the time of the offense). Sensors, traces, and goods can be seen as tangible (e.g., biological material (or fingerprint) of this person is found on, or in the body of the victim).

According to Anderson et al. [33], when evidence is testimonial, one has to consider the basis of the testimonial assertion (inferred, second-hand, or personal knowledge). Besides the testimonial assertion, the credibility of testimonial evidence is determined by the observational sensitivity, objectivity, and veracity of the witness. Observational sensitivity concerns the quality and duration of the observation. Objectivity concerns memory-related issues, like the ability to recall the event and/or whether the witness was biased toward believing the event occurred. In order to score veracity, one has to consider information related to the dishonesty of the source. In addition, a witness statement can be confirmed or contradicted by other statements. One should use the statement of the most credible witness and report the amount of confirming and contradicting witness statements.

The credibility of tangible evidence is determined by its authenticity, accuracy/sensitivity, and reliability [33]. To score authenticity, one has to consider whether documents or recordings are deliberately forged (e.g., forged tickets to provide an alibi) or whether errors were made during the processing of the evidence after it was collected (e.g., DNA was mislabeled during processing). Accuracy/sensitivity concerns the quality of a recording and whether the evidence has the degree of resolution necessary to discriminate between different events (e.g., blurred camera footage only shows a silhouette). Reliability concerns the operating system used to generate the evidence, the operating system is reliable when it gives the same reading on repeated applications (e.g., a medical test showing the same result on all readings when the conditions of the patient were equal).

#### 3.3.3. Adjustment of the Initial Value

In this section, twelve underlying factors were determined that could be used to adjust the initial value of the 24 elements in the library. The elements in the library were carefully selected and their initial score was obtained by the judgement of a large number of experts from the field of homicide investigation. To incorporate the expert judgement in the score, the underlying factors could add or reduce the initial score by a maximum of 50%. The use of a three-point scale to score each individual underlying factor kept the methodology clear and simple, made the scores easily adjustable, and prevented the methodology from becoming overly time-consuming. The three-point scales for all individual underlying factors are displayed in Table 3. After the user has scored the underlying factors, the overall mean score of relevance and credibility is automatically calculated and the initial score is adjusted. When the mean overall scores of both relevance and credibility are more than two, the initial score is increased by 50%. When one has a mean overall score of more than two, and the other has a mean overall score of two, 25% of the initial score is added. When the mean overall score of both relevance and credibility is two, or when one is more than two and one is less than two, the initial score is maintained. The same logic is used to reduce the score when the mean overall score is less than two. The proposed method for determining the influence of the underlying factors ensures an equal likelihood of maintaining, increasing, or reducing the initial score. We believe this method is appropriate, because it does not neglect the expert judgement for the initial score, but also ensures that the score can be adjusted based on case-specific features.

### 3.4. Walk-Through of C-SEL

**Figure 1:**
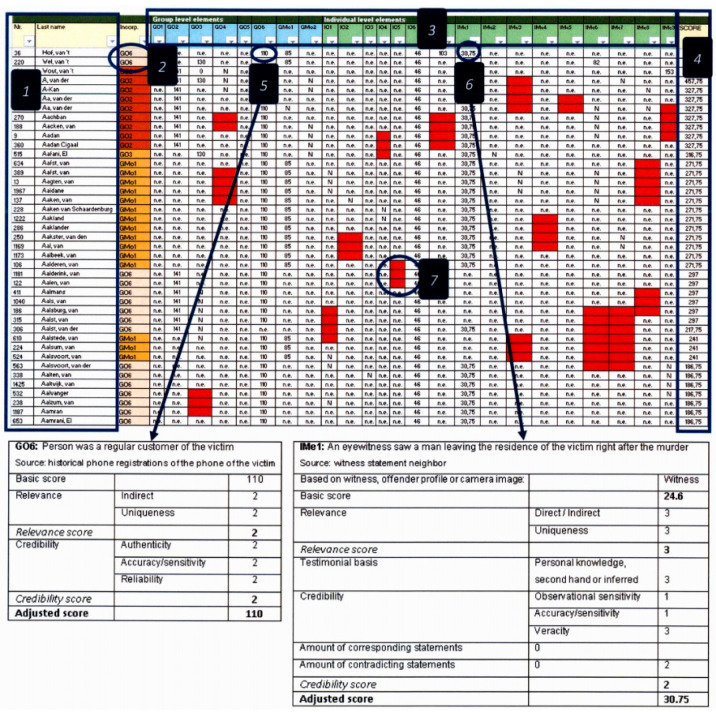
A walk-through of the prototype of the case-specific element library (C-SEL). The first two columns (**1**) are used for identifying information about the incorporated persons of interest (fictitious names are displayed for illustration purposes). The third column (**2**) is used to display the element that was used to incorporate the person of interest. The group and individual level elements are in columns four to twenty-seven (**3**). The last column shows the total score of a person of interest across all elements (**4**). The element that was used to incorporate a person of interest (**5**) is scored on the underlying factors. All other elements and underlying factors (like the person has the same gender as the description of the perpetrator (IMe1) (**6**)) are scored. When a cell is scored ‘Y’, the accompanying score form of the underlying factors appears. After completion, the adjusted score is calculated and displayed. Cells that still need to be scored are red (**7**). When, after investigation, a cell could not be scored as ‘Y’ or ‘N’, it receives the score not established (n.e.) and 20% of the initial score. The screenshots are taken from the crime-related homicide case that will be outlined in the next section.

The elements were selected, an initial value were established, and underlying factors were determined. The result: C-SEL, a case-specific elements library consisting of 24 elements and 12 underlying factors. A walk-through of the process of C-SEL is provided in Figure 1.

## 4. Results: The Evaluation of C-SEL

To evaluate the effectiveness of the C-SEL methodology, three real-world and recently-solved Dutch homicide cases were used: the cases described by Sutmuller et al. [18] to compare the four methodologies that were introduced in the background section (POIPAT, TIE, Rasterfahndung, and ACH). To be able to compare the results with the other four methodologies, the same performance criteria for the collection and prioritization phase were used as in Sutmuller et al. [18].

The performance criterion in the collection phase was met if the name of the perpetrator was incorporated into the investigation in the first two weeks. The performance criterion in the prioritization phase was met if the actual perpetrator was prioritized within the top ten percent of persons of interest. Prioritization is a dynamic process in which each entering person of interest is weighted against the pieces of evidence that are available at that moment. Hence, the performance of the prioritizing phase was measured using the pieces of evidence that were available on the day that the name of the perpetrator appeared in the investigation, weighing all persons of interest that were available on that day [18].

In the next sections, the three cases are shortly introduced, the results of C-SEL on the collection and prioritization of persons of interest are given, and a comparison to the other currently-used methodologies is presented.

### 4.1. Collection

#### 4.1.1. Homicide by Intimate

A corpse was found in the perpetrator’s house. The victim and the perpetrator had a sexual relationship and the perpetrator had previous convictions of manslaughter, violence, and theft. There were no direct eyewitnesses, so there was no description of the perpetrator. The name of the perpetrator appeared in the investigation on the first day, because the murder took place in the residence of the perpetrator. When the group-level elements of C-SEL were applied to this case, the name of the perpetrator could have been incorporated on the first day based on five elements. Three elements concerned the opportunity of the perpetrator: the victim was found dead at the house of the perpetrator (GO2), the perpetrator was seen near the crime scene by an acquaintance (GO3), and the perpetrator had access to the victim by being an extra-marital sexual partner (GO6). A motive for the perpetrator can be deduced from two witnesses (GMo1) and from the criminal history of the perpetrator showing a previous homicide with similar modus operandi (GMo2).

#### 4.1.2. Crime-Related Homicide

The corpse of the victim was found in his own residence. The perpetrator was a drug user and the victim was his regular drug supplier. The distance between the crime scene and the residence of the perpetrator was approximately 75 km and the perpetrator had more than ten previous convictions of property crimes (theft and burglary). An eyewitness saw a 30 to 35-year-old man, 1.80 m tall, with brown curly hair, leaving the residence of the victim right after the murder. The witness also remembered two characters of the license plate of the car the man drove away in. The name of the perpetrator appeared in the investigation on the 21st day, because the perpetrator’s phone number appeared in historical phone registrations of the phone of the victim. When the group-level elements of C-SEL were applied to this case, the name of the perpetrator could have been incorporated based on three elements: a witness described the car the perpetrator left in and remembered two out of six characters of the license plate (GO1), the perpetrator had access to the victim as a regular customer of the victim’s drugs business (GO6), and multiple witnesses stated that the victim had arguments with regular customers (GMo1).

#### 4.1.3. Revenge-Oriented Homicide

A corpse was found in a public place. The victim was a drug user and occasionally robbed people to pay for his addiction. The perpetrator was one of the people that was formerly robbed by the victim. The victim was murdered close to the scene where the former robbery had taken place. The murder scene was approximately three kilometers from the residence of the perpetrator and approximately two kilometers from the residence of the victim. The perpetrator had a previous conviction for threatening a (seemingly random) pedestrian. There were no direct eyewitness, so there was no description of the perpetrator. The name of the perpetrator appeared in the investigation on the 18th day, because a witness told the police about a former robbery connecting the perpetrator and the victim; the perpetrator had reported the robbery, but the police did not link the murder and the robbery prior to the witness making the statement. When the group-level elements were applied to this case, the name of the perpetrator could have been incorporated based on witness statements about robberies committed by the victim, giving the perpetrator a motive (GMo1).

#### 4.1.4. Comparing the Collection Phase to Other Methodologies

In the original case files, the homicide by intimate case was the only one where the perpetrator was incorporated within the first two weeks. In this case, the name of the perpetrator appeared in the investigation on the first day; the crime-related homicide (day 21), and the revenge-oriented homicide (day 18) did not meet the performance criterion. The collection phase was evaluated for two methodologies by Sutmuller et al. [18]. Both TIE and Rasterfahndung managed to incorporate the name of the perpetrator in the investigation within the first two weeks in both the homicide by intimate case and the crime-related homicide, but failed to incorporate the perpetrator in the revenge-oriented homicide. When using C-SEL, the name of the perpetrator would have appeared in the investigation within the first two weeks in all three homicide cases. Besides the fact that C-SEL was able to meet the performance criterion on all three cases, C-SEL incorporated between 250 and 310 persons of interest into the investigation. An approximation of the total number of persons of interest, when the elements of C-SEL and the categories of TIE and Rasterfahndung were used to incorporate persons of interest from the start of the investigation, are displayed in Table 4. C-SEL was more consistent than the other two methodologies in the number of persons of interest that are incorporated. The incorporation of large numbers of persons of interest is one of the downsides of the methodologies currently being used [18]. When the results of the C-SEL methodology are analyzed in more detail, one can see that in all three cases, the name of the perpetrator could have been incorporated based on an element of motive. Both TIE and Rasterfahndung lack categories that incorporate persons of interest based on motive.

### 4.2. Prioritization

#### 4.2.1. Homicide by Intimate

On the day the name of the perpetrator appeared in the investigation (day 1), a total of 13 pieces of evidence were available to prioritize the 71 persons of interest that were present in the investigation. In the prioritization phase, the elements at the group level and the individual level were combined and a ranking of persons of interest was made. In total, 52 people received a score because their identity was registered during the investigation of the crime scene (GO2). In total, 17 people received a score for having free access to the victim, because they were part of the social network of the victim (GO6). There was no one on the list that had an alibi (IO6). The perpetrator scored on one element on the individual level (IO6), but, as mentioned in the previous section, also scored on GO3, GMo1, and GMo2. After the initial score was allocated, the underlying factors were used to adjust the initial score. The perpetrator was the highest prioritized person of interest based on the initial score alone and after adjustment with the underlying factors.

#### 4.2.2. Crime-Related Homicide

On the day the name of the perpetrator appeared in the investigation (day 21), a total of 27 pieces of evidence were available to prioritize the 102 persons of interest that were present in the investigation. The identities of 16 people were registered during the investigation at the crime scene (GO2). Three people visited the victim on the day of the murder (GO3). In total, 63 people had free access to the victim because they were part of his social network (GO6). Seventeen people had a motive derived from a witness statement (GMo1). There was no one on the list that had an alibi (IO6). The last contact of the victim was established (IO7). The list held 68 people with the same gender as the perpetrator, as described by a witness (IMe1). There was one person that owned a similar car as seen by an indirect witness and one owned a similar car as seen by the direct witness (IMe6). One person possessed an item that disappeared from the crime scene (IMe9). The actual perpetrator scored on group-level elements GO1, GO3, and GMo1, and on individual-level elements IO6, IO7, IMe1, and IMe6. The perpetrator also scored on all other features from the description given by the direct witness, IMe2 to IMe5, but because no description of all other people on the list was available, the scores of these elements were not included in his ranking. After the initial score was allocated, the underlying factors were used to adjust the initial score. The perpetrator was the highest prioritized person of interest based on the initial score alone and after adjustment with the underlying factors.

#### 4.2.3. Revenge-Oriented Homicide

On the day the name of the perpetrator appeared in the investigation (day 18), a total of 107 pieces of evidence were available to prioritize the 238 persons of interest that were present in the investigation. In total, 20 people were seen near the crime scene by a witness (GO3). On the list were 104 people that had access to the victim, because they were part of his social network (GO6). For nine people, a motive was derived from a witness statement (GMo1). One witness stated that he heard that a particular person had committed the crime (IO4). None of the people had alibis (IO6). The actual perpetrator scored on group-level element GMo1 and on individual-level element IO6. After the initial score was allocated, the underlying factors were used to adjust the initial score. The perpetrator was prioritized within the top 5.46% of persons of interest based on the initial score alone and within the top 3.36% of persons of interest after adjustment with the underlying factors.

#### 4.2.4. Comparing the Prioritization Phase to Other Methodologies

In Table 5, the percentages in which the perpetrator was prioritized are displayed for C-SEL, based solely on the initial score and after adjustment with the underlying factors as well as the percentages for the four methodologies currently being used. The performance criterion for prioritization was met in the homicide by intimate and crime-related homicide cases by all four methodologies in Sutmuller et al. [18], but all four methodologies failed to meet the performance criterion in the revenge-oriented homicide. When C-SEL was used to prioritize, the perpetrator was prioritized within the top ten percent in all three cases. In the revenge-oriented homicide, the adjustment of the initial score by the underlying factors caused the perpetrator to be prioritized within the top 3.36%, instead of within the top 5.46% based on the initial score alone. Although both the initial score of C-SEL and the adjusted C-SEL score met the performance criterion, this is an indication that the adjustment by the underlying factors can add value to C-SEL. When the results of the five methodologies are analyzed in more detail, one can see that, again, the inclusion of elements of motive made the difference. The initial score and adjustment of GMo1 caused the perpetrator in the revenge-oriented homicide to be prioritized within the top 3.36%.

## 5. Discussion

In this paper, we developed and evaluated C-SEL, a methodology that can be used to build case-specific tools that assist criminal investigators with the incorporation and prioritization of persons of interest in homicide cases. In this section, the results, implications, and the limitations of our research are discussed in more detail and opportunities for future research are outlined.

### 5.1. Developing C-SEL

This research started with the development of a new methodology, 24 elements, and 12 underlying factors became C-SEL. The selection of elements, the allocation of an initial score, and the determination of underlying factors were based on the literature and expert judgement. A common limitation of using experts is that they bring their own mindset and experience. To overcome this bias in the allocation of the initial value of the elements, three different groups of experts within the field of criminal investigation were selected.

The large number of expert participants from the field and the large degree of agreement between the groups supported the idea that the initial score of the elements can be used in practice. However, the participants had to make a forced choice between the elements, without being able to indicate the difference in value between the elements. This limitation for the participants could have contributed to the degree of agreement.

The use of a three point scale and the arithmetical properties on how the underlying factors and the initial scores interact are also based on expert judgement. These choices, like all choices made in the development of a methodology, are debatable. So, the effectiveness of C-SEL to incorporate and prioritize persons of interest in homicide cases was evaluated using three real-world Dutch homicide cases. To put the results of C-SEL into perspective, a comparison was made with four methodologies that are currently being used.

### 5.2. Case Study

The results of C-SEL can be seen as promising: in all three cases, the name of the perpetrator was incorporated into the investigation within two weeks and the perpetrator was prioritized within the top ten percent of persons of interest. These results are better than the results of the four other methodologies. However, the limited number of cases makes it hard to draw conclusions about the superiority of the methodology over the others.

Historical real-world cases were used in which a suspect was successfully prosecuted. All three were Dutch single-offender and single-victim homicides. With closed cases, it is impossible to steer the gathering and processing of information. This might have affected the performance of methodologies. However, because C-SEL is evaluated using the same information as the four methodologies from Sutmuller et al. [18], we believe this limitation is acceptable. We used Dutch homicide cases to evaluate C-SEL, but because the universal OMM-topology is used in the development of the methodology, we believe that the methodology could be used in other countries, and future research should be conducted to investigate whether C-SEL can be used globally.

Before C-SEL can be used in practice, more cases are needed to further validate its effectiveness. The collection of pieces of evidence in ongoing cases is dynamic and the relevance and credibility of pieces of evidence can change over time. C-SEL is able to deal with changing elements and underlying factors, but future research should be conducted on how C-SEL works in ongoing investigations. Other cases that should be used to further validate the effectiveness of C-SEL are cold cases and complex cases like homicides with multiple offenders, homicides with thousands of persons of interest, or homicides that took a long time to solve. Given the positive results, it would be interesting to investigate the application of the C-SEL methodology to other types of severe crimes like robbery or rape.

One of the problems faced by homicide investigators in the digital era is weighing the vast amount of diverse evidence becoming available. The elements of C-SEL aim to cover the whole range of evidence in this digital era. In the used cases, all pieces of evidence could have been accommodated by the elements of C-SEL. Whether the elements of C-SEL could cover all types of evidence in the digital era should be the focus of future research. One difference between the four previously evaluated methodologies and C-SEL is the inclusion of elements of motive. These elements received the highest initial score by all three groups of experts. The inclusion of the elements of motive had a positive effect on the results of C-SEL in comparison to the four other methodologies. Whether the elements of motive are important in all types of homicide could be the subject of future research.

The adjustment of the initial score seems to have added value in the prioritization phase of the revenge-oriented homicide. The consideration of the relevance and credibility of the source caused the perpetrator to be prioritized higher, but both the initial and adjusted scores of C-SEL met the performance criterion. Future research should be conducted to investigate the added value of the underlying factors. Despite the added value of the underlying factors needing to be confirmed in future research, we believe that the possibility of adjusting the initial weight, based on the relevance and credibility of the source, is necessary. A certain piece of evidence could identify the perpetrator in one case and could be worthless in another. The scoring of underlying factors for the elements was made as simple as possible. However, the process of scoring all these underlying factors could be potentially burdensome. The scoring of the underlying factors is done by the end-user of the methodology. We believe that the end-user of C-SEL should be a member of the investigative team that is in charge of steering the investigation based on information (like the Dutch crime analyst). Future research should be conducted to investigate the time needed to use C-SEL during an ongoing investigation, the extent to which end-users agree (or differ) on scoring the underlying factors, and the degree of training needed to use C-SEL.

Another problem faced by homicide investigators in the digital era is the incorporation of a large number of persons of interest. C-SEL incorporated between 250 and 310 persons of interest. This seems to be a workable number of persons of interest. Future research should be conducted to investigate whether this number of persons of interest is stable across more cases. We believe that it is necessary, in cases where the perpetrator is not caught red-handed, to maintain an overview and pursue more potential suspects. Suspect-driven investigations, which focus on pursuing one suspect, could facilitate tunnel vision [38]. C-SEL pursues multiple persons of interest and seems to be able to prioritize the actual perpetrator within the top ten percent of persons of interest. Future research should be conducted to determine whether C-SEL effectively reduces tunnel vision.

Since it was not the aim of this study, C-SEL does not address the last phase of handling persons of interest in homicide cases: the elimination of persons no longer of interest. Sutmuller et al. [18] showed that the elimination of persons of interest from the investigation is difficult. The aim of a homicide investigation is to bring a suspect to trial, and although the elimination of all other persons of interest is the best way to reach that goal, this is often impossible due to capacity and workload in homicide investigation. One possibility of overcoming this problem is to search for and upgrade the value of an element to the extent that a person of interest that scores on that element must be the perpetrator. When such an element is found (e.g., the sperm of an unknown man on a female victim in a sexually motivated homicide case), the investigation can be focused by eliminating persons of interest based on that element. Whether C-SEL is able to appoint such elements, based on their initial or adjusted score, could be the focus of future research.

## 6. Conclusions

One of the challenges the police are now facing is the increasing availability of data. In homicide investigations, the processing of all persons of interest and pieces of evidence becomes a daunting task and this could contribute to the increasing percentage of unsolved homicide cases [3,4,5,6,7]. This paper introduces a new methodology (C-SEL) that can be used to build a case-specific tool that assists criminal investigators with the incorporation and prioritization of persons of interest in homicide cases in the digital era. The methodology was primarily developed through expert judgement and the development consisted of three stages: the selection of elements, the valuation of elements, and the determination of underlying factors. The effectiveness of C-SEL was evaluated in a case study using three recent real-world Dutch homicide cases. C-SEL incorporated between 250–310 persons of interest and the name of the perpetrator would have been incorporated into the investigation within the first two weeks in all three cases. C-SEL made it possible to determine the relevance and credibility of the source of the elements and in all three cases, the perpetrator was prioritized within the top four percent of persons of interest. The results obtained with the C-SEL methodology were compared to four methodologies that are currently used in practice. The application of C-SEL showed better results than when applying the other four methodologies. We believe that the introduced C-SEL methodology is a promising first step to deal with the increasing number of persons of interest and pieces of evidence in the digital era.

## Figures and Tables

**Table 1:** The proposed library of elements that can be used to build a case-specific incorporation and prioritization tool in homicide cases. The library is structured using group (G) and individual (I) levels and the opportunity (O), motive (Mo), and means (Me) topology (OMM).

Level	OMM	Description of Element
G	O1	Persons whose goods (for example, a mobile device and/or vehicle) were registered by a sensor that provides coverage at the crime scene at the time of the offense
G	O2	Persons whose identity or good is registered during the investigation at the crime scene
G	O3	Persons of whom a witness states that they were near the crime scene at the time of the offense
G	O4	Persons visible on camera footage near the crime scene at the time of the crime
G	O5	Persons who live, work or recreate in an area that is determined in a behavioral offender profile
G	O6	Persons who have free access to the victim because they are part of the social network of the victim
G	Mo1	Persons who have a motive derived from a witness’s statement
G	Mo2	Persons who have a motive derived from a behavioral offender profile or the life story of the victim
G	Me1	Persons who meet the description given by a witness
G	Me2	Persons who meet the description obtained by camera image
G	Me3	Persons who meet the profile that has been put forward in a behavioral offender profile
G	Me4	Persons who may have access to the same type of murder weapon used by the perpetrator
G	Me5	Persons who can be linked to a good (other than the murder weapon) that is (partially) left behind, seen or recorded at the crime scene (for example clothing or tools)
I	O1	Biological material (or fingerprint) of this person is found on or in the body of the victim
I	O2	Biological material (or fingerprint) of this person is found on the clothing and/or in the immediate vicinity of the victim
I	O3	A witness states to have observed that this person committed the crime
I	O4	A witness states to have heard or suspects that this person has committed the crime
I	O5	A camera captures this person during the execution of the crime
I	O6	This person has no alibi
I	O7	This person is the victim’s last established contact
I	Mo1	Police information or antecedents show that this person has a motive
I	Mo2	A testimony from a witness shows that this person has a motive
I	Me1	Person has the same gender as the description of the perpetrator
I	Me2	Person has the same race as the description of the perpetrator
I	Me3	Person has the same height as the description of the perpetrator
I	Me4	Person is the same age as the description of the perpetrator
I	Me5	Person has the same clothing as the description of the perpetrator
I	Me6	Person has the same means of transport as the perpetrator
I	Me7	Person has a good (other than the murder weapon) that is (partially) left behind at the crime scene (for example, clothing where fibers have been found or tools for which traces have been found)
I	Me8	Person has the murder weapon that has been used according to comparative research
I	Me9	Person has a good that has disappeared from the crime scene

**Table 2:** The mean score (standard deviation) of the survey results for the three expert groups (CA = Crime Analyst; MCI = Master of Criminal Investigation; IP = Investigative Psychologist) and the calculated initial scores for the elements. The displayed initial scores for features of a description (IMe 1, IMe2, IMe3, IMe4, IMe5 and IMe6) are based on a description obtained from camera footage: 60% of this score is allocated when the source of the description is a witness statement and 57% of this score is allocated when the source of the description is an offender profile. * indicates that the element was included in the final version of the library.

Description	CA	MCI	IP	Initial Score	In Library
Opportunity	1.92 (0.76)	1.95 (0.73)	1.89 (0.69)	-	-
Motive	1.51 (0.77)	1.55 (0.86)	1.46 (0.79)	-	-
Means	2.57 (0.55)	2.50 (0.55)	2.64 (0.49)	-	-
GO1	3.59 (1.38)	3.63 (1.51)	3.82 (1.33)	116	*
GO2	3.00 (1.51)	3.14 (1.66)	2.46 (1.19)	141	*
GO3	3.05 (1.39)	3.05 (1.29)	3.82 (1.19)	130	*
GO4	2.05 (1.15)	2.07 (0.97)	2.00 (0.94)	171	*
GO5	5.22 (0.98)	5.50 (0.92)	5.14 (1.04)	59	*
GO6	4.08 (1.89)	3.62 (1.70)	3.75 (1.84)	110	*
GMo1	1.51 (0.51)	1.60 (0.50)	1.86 (0.36)	170	*
GMo2	1.49 (0.51)	1.40 (0.50)	1.14 (0.36)	204	*
GMe1	3.41 (1.09)	3.50 (1.06)	3.82 (0.98)	70	
GMe2	1.92 (0.92)	1.71 (0.77)	2.11 (0.79)	118	
GMe3	3.51 (1.19)	3.90 (0.91)	3.54 (1.17)	67	
GMe4	4.24 (0.98)	4.10 (1.12)	3.93 (1.36)	55	
GMe5	1.92 (1.19)	1.79 (0.95)	1.61 (0.92)	121	
IO1	2.84 (1.19)	2.38 (1.01)	2.36 (0.68)	162	*
IO2	4.05 (0.94)	4.12 (1.19)	3.86 (0.93)	118	*
IO3	2.89 (1.33)	2.95 (1.13)	3.57 (1.43)	146	*
IO4	5.95 (1.10)	5.93 (0.87)	6.00 (0.90)	61	*
IO5	1.57 (1.41)	1.52 (1.04)	1.04 (0.19)	196	*
IO6	6.16 (1.44)	6.62 (0.66)	6.57 (0.69)	46	*
IO7	4.54 (1.30)	4.48 (1.23)	4.61 (0.92)	103	*
IMo1	1.46 (0.51)	1.55 (0.50)	1.32 (0.48)	192	
IMo2	1.54 (0.51)	1.45 (0.50)	1.68 (0.48)	182	
IMe1	7.30 (1.58)	7.62 (1.74)	7.43 (1.69)	41	*
IMe2	7.19 (1.31)	7.14 (1.18)	7.25 (1.21)	45	*
IMe3	6.86 (1.70)	7.21 (1.24)	6.79 (1.23)	48	*
IMe4	6.89 (1.61)	6.86 (1.35)	7.29 (1.30)	48	*
IMe5	5.41 (1.67)	5.12 (1.35)	5.32 (1.56)	76	*
IMe6	4.95 (1.35)	4.88 (1.53)	4.75 (1.62)	82	*
IMe7	2.32 (0.75)	2.50 (0.74)	2.68 (0.77)	120	*
IMe8	1.59 (1.57)	1.38 (0.91)	1.21 (0.57)	137	*
IMe9	2.49 (0.87)	2.29 (0.67)	2.29 (0.60)	122	*

**Table 3:** The underlying factors of relevance and credibility, the three-point scale to score these factors, and the elements that use the underlying factor are displayed.

**Relevance**	**Three-Point Scale**	**Used by Element**
Direct/indirect	1 = Makes person of interest; 2 = Makes person suspect; 3 = Makes person perpetrator	All
Uniqueness	1 = Not very unique; 2 = Neutral; 3 = Very Unique	All
Location	1 = Wide crime scene (<100 m); 2 = Crime scene (<20 m); 3 = Contact with victim (<5 m)	GO: 1,2,3,4
Time	1 = Day of the homicide; 2 = Within hour of the homicide; 3 = Within fifteen minutes of the homicide	GO: 1,2,3,4
**Credibility (testimonial)**	**Three-point scale**	**Used by element**
Testimonial basis	1 = Inferred; 2 = Second hand; 3 = Personal knowledge	GO: 3,5,6 GMo: 1,2 IO: 3,4,6,7 IMe: 1,2,3,4,5,6,7
Observational sensitivity	1 = Problematic; 2 = Neutral; 3 = Good	GO: 3,6 GMo: 1 IO: 3,4,6,7 IMe: 1,2,3,4,5,6,7
Objectivity	1 = Problematic; 2 = Neutral; 3 = Good	GO: 3,6 GMo: 1 IO: 3,4,6,7 IMe: 1,2,3,4,5,6,7
Veracity	1 = Problematic; 2 = Neutral; 3 = Good	GO: 3,6 GMo: 1 IO: 3,4,6,7 IMe: 1,2,3,4,5,6,7
Contradicting and confirming	1 = Contradicting > confirming; 2 = Contradicting = confirming; 3 = Contradicting < confirming	GO: 3,6 GMo: 1 IO: 3,4,6,7 IMe: 1,2,3,4,5,6,7
**Credibility (tangible)**	**Three-point scale**	**Used by element**
Authenticity	1 = (Possibly) not authentic; 2 = Neutral; 3 = Authentic	GO: 1,2,4,6 GMo: IO: 1,2,5,6,7 IMe: 1,2,3,4,5,6,7,8,9
Accuracy/sensitivity	1 = Bad quality; 2 = Neutral; 3 = Good quality	GO: 1,2,4,6 GMo: IO: 1,2,5,6,7 IMe: 1,2,3,4,5,6,7,8,9
Reliability	1 = Not reliable; 2 = Neutral; 3 = Reliable	GO: 1,2,4,6 GMo: IO: 1,2,5,6,7 IMe: 1,2,3,4,5,6,7,8,9

**Table 4:** Summary of results from the collection phase showing the approximate maximum number of persons of interest when using the case-specific element library (C-SEL), Trace Investigate Evaluate (TIE), and Rasterfahndung. * indicates that the perpetrator was in this group. Approximations were made for element GO1, based on Offermans, Priem, and Tennekes [36] and element GO6, based on van Asselt-Goverts, Embregts, Hendriks, Wegman, and Teunisse [37]. For the other elements, the number of persons of interest in the complete case file that met these elements was used. The approximations for TIE and Rasterfahndung were taken from Sutmuller et al. [18].

Methodology	Homicide by Intimate	Crime-Related Homicide	Revenge-Oriented Homicide
C-SEL	275 *	253 *	309 *
TIE	10.317 *	209.606 *	22.933
Rasterfahndung	191 *	173.336 *	191

**Table 5:** Summary of percentages in which the perpetrator is prioritized by the initial score of the case-specific element library (C-SEL IS) alone and after adjustment of the underlying factors (C-SEL AA) as well as Trace Investigate Evaluate (TIE), Person of Interest Priority Assessment Tool (POIPAT), Rasterfahndung, and Analysis of Competing Hypotheses (ACH). The percentages for TIE, POIPAT, Rasterfahndung, and ACH were taken from Sutmuller et al. [18].

Methodology	Homicide by Intimate	Crime-Related Homicide	Revenge-Oriented Homicide
C-SEL IS	1%	1%	5.46%
C-SEL AA	1%	1%	3.36%
TIE	1%	4.90%	17.23%
POIPAT	1%	3.92%	58.40%
Rasterfahndung	1%	4.90%	17.23%
ACH	1%	8.82%	86.55%
